# Interventional Treatment vs Conservative Management of Unruptured Brain Arteriovenous Malformations

**DOI:** 10.1001/jamanetworkopen.2025.43408

**Published:** 2025-11-13

**Authors:** Heze Han, Yu Chen, Li Ma, Hengwei Jin, Dezhi Gao, Zhipeng Li, Ruinan Li, Haibin Zhang, Kexin Yuan, Anqi Li, Tengfei Yu, Qinghui Zhu, Chengzhuo Wang, Yukun Zhang, Hongwei Zhang, Debin Yan, Xiaofeng Chao, Zhengfeng Lin, Youxiang Li, Shibin Sun, Yuanli Zhao, Xiaolin Chen, Shuo Wang

**Affiliations:** 1 Department of Neurosurgery, Beijing Tiantan Hospital, Capital Medical University, Beijing, China; 2 Beijing Neurosurgical Institute, Beijing Tiantan Hospital, Capital Medical University, Beijing, China; 3 China National Clinical Research Center for Neurological Diseases, Beijing, China; 4 Department of Neurological Surgery, University of Pittsburgh Medical Center, Pittsburgh, Pennsylvania; 5 Department of Interventional Neuroradiology, Beijing Tiantan Hospital, Capital Medical University, Beijing, China; 6 Department of Gamma-Knife Center, Beijing Tiantan Hospital, Capital Medical University, Beijing, China; 7 Department of Neurosurgery, Peking Union Medical College Hospital, Chinese Academy of Medical Sciences and Peking Union Medical College, Beijing, China; 8 Department of Neurosurgery, Peking University International Hospital, Peking University, Beijing, China; 9 Department of Neurosurgery, Shanxi Provincial People’s Hospital, Shanxi, China; 10 Department of Neurosurgery, The Second Affiliated Hospital of Xuzhou Medical University, Jiangsu, China; 11 Department of Neurosurgery, The First People’s Hospital of Qinzhou, Guangxi, China

## Abstract

**Importance:**

The optimal management of unruptured brain arteriovenous malformations (AVMs) remains controversial, as the risk-benefit balance between interventional treatment and conservative management is unclear. While ARUBA (A Randomized Trial of Unruptured Brain AVMs) favored conservative management in the short term, methodologic limitations and limited follow-up have left uncertainty regarding long-term outcomes.

**Objective:**

To evaluate the association between interventional treatment and hemorrhage-free survival, compared with conservative management, in patients with unruptured AVMs.

**Design, Setting, and Participants:**

This cohort study used a target trial emulation approach based on data from the Multimodality Treatment for Brain Arteriovenous Malformation in Mainland China (MATCH) registry, a nationwide, multicenter prospective collaboration conducted from August 1, 2011, to December 31, 2021. Patients with unruptured AVMs at baseline were included. Data analyses were conducted from January 24 to April 6, 2025.

**Exposures:**

Interventional treatment (microsurgical resection, stereotactic radiosurgery, endovascular embolization, or combinations thereof) initiated within 6 months of diagnosis vs conservative management (observation and medical therapy).

**Main Outcomes and Measures:**

The primary outcome was 5-year hemorrhage-free survival, defined as survival without AVM-related hemorrhage or death. The secondary outcome was 10-year hemorrhage-free survival. Hazard ratios (HRs) and risk differences were estimated using inverse probability of censoring-weighted survival analyses following a clone-censor-weight approach.

**Results:**

Among the 1770 patients included in the analysis (median age, 26.2 [IQR, 16.5-37.6] years; 1059 [59.8%] male), the estimated 5-year hemorrhage-free survival was 96.23% (95% CI, 93.95%-97.65%) for the interventional treatment group and 89.00% (95% CI, 86.37%-91.24%) for the conservative management group, yielding a risk difference of 7.23% (95% CI, 4.78%-9.91%) and an HR for hemorrhage of 0.44 (95% CI, 0.33-0.57). At 10 years, the risk difference was 8.37% (95% CI, 2.68%-15.70%) and the HR was 0.56 (95% CI, 0.42-0.69). Subgroup analyses indicated that the benefit of the intervention was not observed in patients with high-grade AVMs or diffuse nidus, for whom outcomes were similar to those of patients with conservative management. Only microsurgical resection was consistently associated with reduced hemorrhage risk in sensitivity analyses.

**Conclusions and Relevance:**

In this nationwide cohort study using target trial emulation, interventional treatment of unruptured AVMs was associated with improved hemorrhage-free survival at 5 and 10 years. These findings support individualized decision-making in the management of unruptured AVMs and demonstrate how target trial emulation can inform clinical questions where randomized clinical trials are not feasible.

## Key Points

**Question:**

Is interventional treatment for unruptured brain arteriovenous malformations (AVMs) associated with improved hemorrhage-free survival compared with conservative management?

**Findings:**

In this cohort study that included 1770 patients with unruptured AVMs, interventional treatment was associated with significantly higher estimated hemorrhage-free survival at 5 years (96% vs 89%) and 10 years (84% vs 76%) compared with conservative management.

**Meaning:**

These findings suggest that interventional treatment may reduce long-term hemorrhage risk in patients with unruptured AVMs.

## Teaser

This cohort study uses a target trial emulation approach to compare hemorrhage-free survival among patients receiving interventional treatment vs conservative management for unruptured brain arteriovenous malformations (AVMs).

## Introduction

Unruptured brain arteriovenous malformations (AVMs) present a challenging treatment dilemma in neurosurgery.^1^ These vascular lesions carry a lifelong risk of intracranial hemorrhage, often resulting in stroke or death, particularly in younger patients.^2^ However, all available treatments—including microsurgery, stereotactic radiosurgery (SRS), and embolization—carry procedural risks.^3^ Advances in neuroimaging have increased the incidental detection of unruptured AVMs, forcing clinicians to weigh preventive treatment risks against the natural risk of rupture. Consequently, the optimal management strategy for unruptured AVMs remains uncertain and highly debated.^4^

This uncertainty was highlighted by ARUBA (A Randomized Trial of Unruptured Brain AVMs), the only randomized clinical trial comparing intervention with conservative management for AVMs.^5^ ARUBA suggested a short-term benefit for conservative management, but several critical limitations have raised questions about its generalizability.^6^ Subsequent observational studies^7,8,9,10,11,12,13^ have reported mixed results, reflecting methodologic issues such as selection bias, confounding, and immortal time bias. Thus, it remains unclear whether the reported superiority of an intervention or conservative management in observational studies reflects a true effect or methodologic artifacts.

Given the rarity of AVMs and the low incidence of hemorrhage, conducting another large-scale randomized clinical trial with sufficient power and long-term follow-up is challenging. In this context, target trial emulation has emerged as a robust approach for deriving causal estimates from observational data.^14,15^ By explicitly emulating the design and analysis of an ideal randomized clinical trial, this method can reduce common biases and more accurately reflect clinical outcomes.^16,17^ In the present study, we applied a target trial emulation approach using data from a nationwide, multicenter prospective registry to evaluate the association of interventional treatment vs conservative management with hemorrhage-free survival in patients with unruptured AVMs, aiming to infer the causal effect and to provide reliable evidence to guide neurosurgical decision-making.

## Methods

### Study Setting and Data Sources

This cohort study emulated a hypothetical target trial to compare interventional treatment with conservative management in patients with unruptured AVMs. We used individual-level data from the Multimodality Treatment for Brain Arteriovenous Malformation in Mainland China (MATCH) registry, a nationwide, multicenter prospective collaboration conducted between August 1, 2011, and December 31, 2021.^18^ The institutional review board of Beijing Tiantan Hospital approved registry use, and all patients provided written informed consent at hospital admission. This study follows the Strengthening the Reporting of Observational Studies in Epidemiology (STROBE) reporting guideline.

We included patients diagnosed with unruptured AVMs at baseline. Exclusions were based on (1) missing essential clinical or imaging data or (2) hemorrhage leading to AVM diagnosis. Further details on data quality management are provided in eMethods 1 in Supplement 1.

### Hypothetical Target Trial

We specified the protocol of the hypothetical trial that our analysis aimed to emulate. Eligible patients would be randomly assigned to intervention (microsurgery, embolization, or SRS, alone or combined) or conservative treatment (observation and medical therapy). All participants would be followed up from randomization until hemorrhage, death, or end of follow-up. The primary outcome was hemorrhage-free survival. The causal estimands were the intention-to-treat and per-protocol effects of interventional treatment vs conservative management. This hypothetical trial would resemble a pragmatic, open-label design comparing the 2 strategies using absolute risk differences and hazard ratios (HRs) for 5-year hemorrhage-free survival. A detailed specification of the protocol and its emulation using observational data are provided in eTable 1 in Supplement 1.

### Emulated Trial Design

#### Treatment Strategies

We compared 2 management approaches reflecting a pragmatic interventional treatment vs a conservative management strategy for unruptured AVMs. Interventional treatment was defined as initiation of any procedure to obliterate the AVM, while conservative care referred to observation and medical therapy for symptoms. In the target trial, patients would be randomized at diagnosis with therapy initiated shortly thereafter. In our emulation, a 6-month grace period after diagnosis was allowed for initiating intervention. Patients who did not begin treatment within this period were considered to remain in the conservative management group. Thereafter, participants were expected to continue with their assigned strategy.

#### Target Trial Emulation

We used the clone-censor-weight (CCW) method to estimate causal effects consistent with the target-trial design.^19^ This approach addresses key biases in observational data, such as baseline confounding and immortal time bias.^14,20^ We cloned each eligible patient into 2 identical records, assigning one clone to the intervention group and the other clone to the conservative management group. Time zero was the date of AVM diagnosis. Interventional treatment clones were censored at 6 months if untreated; conservative management clones were censored immediately before any intervention. The treatment patterns and censoring rules are illustrated in eFigures 1 and 2 in Supplement 1. While cloning balances baseline characteristics, censoring can introduce selection bias. To adjust for this, we calculated inverse probability of censoring weights (IPCW),^21^ estimating the probability of remaining uncensored at each time point. Details for the weighting procedure are in eMethods 2 in Supplement 1. By applying the CCW method, we estimated the per-protocol effect of interventional treatment vs conservative management as for a randomized clinical trial.^22^

#### Outcomes and Follow-Up

The primary outcome was 5-year hemorrhage-free survival, defined as absence of intracranial hemorrhage or death within 5 years of diagnosis. Hemorrhagic stroke or death was attributed to AVM rupture, confirmed by imaging or clinical documentation. Patients alive and free of hemorrhage at 5 years were censored at that time. The secondary outcome was 10-year hemorrhage-free survival. Follow-up concluded at the earliest occurrence of AVM-related hemorrhage, AVM-related death, last recorded contact, or 10 years after diagnosis. Outcomes were assessed using absolute differences in event rates and HRs, with 95% CIs. Outcomes were evaluated through telephone interviews or medical record reviews conducted by clinical research coordinators at 3-month intervals initially, annually for the first 3 years, and every 5 years thereafter. To minimize follow-up bias, strategies to promote participant retention were implemented (eMethods 3 in Supplement 1).

#### Covariates

Baseline variables included demographic characteristics (age and sex), clinical presentations (seizures, headaches, and neurologic deficits), and AVM radiographic features.^23^ All imaging features were extracted from preintervention scans and reviewed by neurosurgery residents trained by senior neuroradiologists (H.J. and Y.L.). Missing imaging data were imputed using the missForest package in R software, version 4.4.1. These covariates were selected for their potential influence on treatment decisions and outcomes. To evaluate covariate balance between groups, we calculated standardized mean differences at the end of the 6-month grace period. Covariates with absolute standardized mean differences below 0.1 were considered well balanced.

### Statistical Analysis

Outcome incidence was expressed as events per 100 person-years, with person-time calculated from AVM diagnosis to outcome, censoring, or end of follow-up. The primary analysis compared hemorrhage-free survival between groups using IPCW survival analysis. The nonparametric Kaplan-Meier estimator generated cumulative hazard curves using these weights, and absolute risk differences at 5 and 10 years were estimated. We also plotted the unweighted Kaplan-Meier curves and curves according to receipt of actual treatment for comparison. To account for uncertainty in weighting and potential inflation of sample size due to cloning, we performed nonparametric bootstrapping of the CCW procedure with 200 replicates to derive valid 95% CIs. In addition to the main analyses, we performed an exploratory nested case-control study within the emulated cohort to investigate baseline factors associated with intracranial hemorrhage (eMethods 4 in Supplement 1). All analyses were conducted using R, version 4.4.1 (R Program for Statistical Computing) between January 24 and April 6, 2025. Two-sided *P* < .05 indicated statistical significance.

Prespecified subgroup analyses evaluated whether estimated treatment effects varied across key patient characteristics. Analyses were stratified by age (<18 vs ≥18 years), Spetzler-Martin (S-M) grade (low surgical risk: 1-2; intermediate surgical risk: 3; and high surgical risk: 4-5), Lawton-Young grade (low surgical risk: 2; intermediate surgical risk: 3; high surgical risk: 4-5), ventricular system involvement, venous aneurysm, deep location, and exclusively deep drainage (VALE) risk score (low: −4 to −3; moderate: −2 to 1; high: 2-5), combined S-M grade (1-3 vs 4-5), combined VALE score (−4 to 1 vs 2-5), and angiographic features (eloquence region, deep location, feeding artery aneurysm, diffuse nidus, deep drainage, and venous aneurysm). For each subgroup, IPCW weights were reestimated and outcome analysis was repeated.

We conducted several sensitivity analyses to assess robustness, including altering the grace period, analyzing outcomes by single treatment modality, restricting to ARUBA-eligible patients, excluding patients with missing data, applying propensity score matching, and redefining the outcome to include functional impairment. Detailed descriptions are provided in eMethods 5 in Supplement 1.

## Results

### Baseline Characteristics

**Figure 1.**
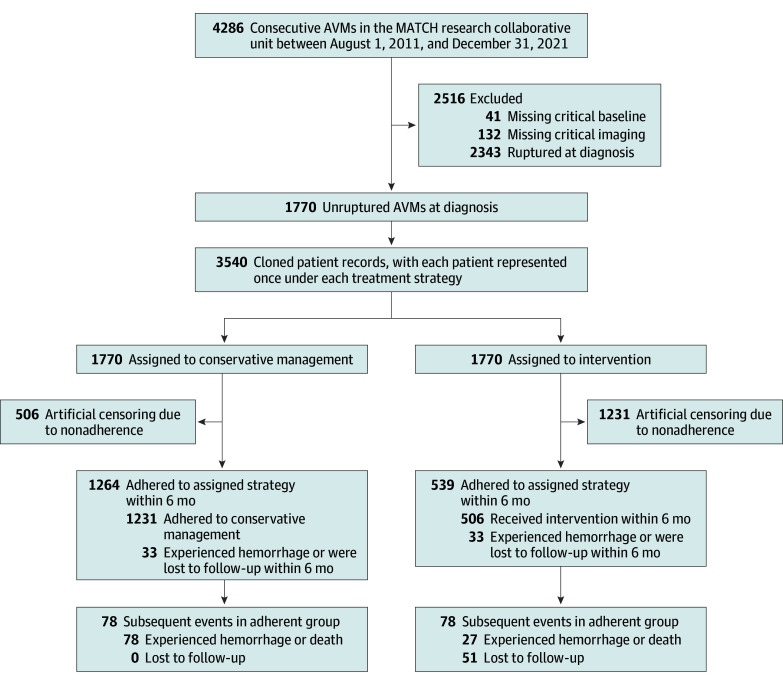
Flowchart of the Study Population AVM indicates arteriovenous malformation; MATCH, Multimodality Treatment for Brain Arteriovenous Malformation in Mainland China.

A total of 1770 patients with unruptured AVMs met eligibility criteria and were included in the target trial emulation. The distribution of missing values is shown in eTable 2 in Supplement 1. The median follow-up in the original cohort was 7.02 (IQR, 3.85-10.40) years. The flowchart of patient inclusion and treatment allocation is shown in Figure 1. Of these patients, 506 (28.6%) received interventional treatment within the 6-month grace period, 1231 (69.5%) received conservative management, and 33 (1.9%) experienced hemorrhage or were lost to follow-up within 6 months. Baseline demographic characteristics and AVM characteristics are summarized in Table 1. The median patient age was 26.2 (IQR, 16.5-37.6) years; 711 patients (40.2%) were female and 1059 (59.8%) were male. Treatment strategy by the ultimate intervention status is described in eTable 3 in Supplement 1. Patients undergoing conservative management during the grace period were generally younger and had more complex AVMs, including higher S-M grades, larger nidus size, diffuse architecture, deep perforator supply, single draining vein, ventricular involvement, and eloquent location, compared with those who underwent the intervention.

**Table 1.** Baseline Patient Characteristics

Characteristic	No. (%) (N = 1770)
Sex	
Male	1059 (59.8)
Female	711 (40.2)
Age at diagnosis, median (IQR), y	26.2 (16.5-37.6)
Clinical presentation	
Asymptomatic	141 (8.0)
Seizure	727 (41.1)
Headache	569 (32.1)
Neurological deficit	339 (19.2)
Spetzler-Martin grade^a^	
1	253 (14.3)
2	554 (31.3)
3	595 (33.6)
4-5	368 (20.8)
Lawton-Young grade^a^	
2	371 (21.0)
3	778 (44.0)
4-5	621 (35.1)
VALE score	
–4 to –3 (low risk)	315 (17.8)
–2 to 1 (moderate risk)	983 (55.5)
2-5 (high risk)	472 (26.7)
AVM location	
Ventricular system involvement	643 (36.3)
Frontal	538 (30.4)
Parietal	527 (29.8)
Temporal	519 (29.3)
Occipital	390 (22.0)
Basal ganglia	127 (7.2)
Thalamus	65 (3.7)
Cerebellum	124 (7.0)
Brainstem	49 (2.8)
Exclusively deep location	277 (15.6)
Infratentorial location	159 (9.0)
Eloquent region^b^	948 (53.6)
AVM size, cm	
<3	595 (33.6)
3-6	900 (50.8)
>6	275 (15.5)
Feeding artery	
Feeding artery dilatation	1145 (64.7)
Single feeding artery	301 (17.0)
Multiple-source supply	656 (37.1)
Deep perforator supply	558 (31.5)
Feeding artery aneurysm	264 (14.9)
Diffuse nidus	422 (23.8)
Drainage vein	
Exclusively deep drainage	198 (11.2)
Single vein drainage	742 (41.9)
Venous stenosis	207 (11.7)
Vein aneurysm	534 (30.2)

Abbreviations: AVM, arteriovenous malformation; VALE, ventricular system involvement, venous aneurysm, deep location, and exclusively deep drainage.

^a^ Higher grades indicate greater surgical complexity and risk.

^b^ Defined as sensory, motor, language, or visual cortex; hypothalamus or thalamus; internal capsule; brain stem; cerebellar peduncles; and deep cerebellar nuclei.

After applying the CCW approach with IPCW, baseline covariates between both treatment groups were well balanced, effectively simulating randomization (eFigure 3 in Supplement 1). A detailed breakdown of treatment modalities within both groups is provided in eTable 4 in Supplement 1. A total of 211 of 539 patients in the intervention group (39.1%) underwent microsurgery, either alone or in combination with other modalities. To further clarify patient selection for microsurgical treatment, baseline characteristics of patients treated with microsurgery vs those treated without microsurgery in the interventional treatment group are presented in eTable 5 in Supplement 1. In general, patients undergoing microsurgery were more likely to have seizures, a low S-M grade, a low VALE score, and frontal location.

### Primary Analysis of the Target Trial Emulation

**Figure 2.**
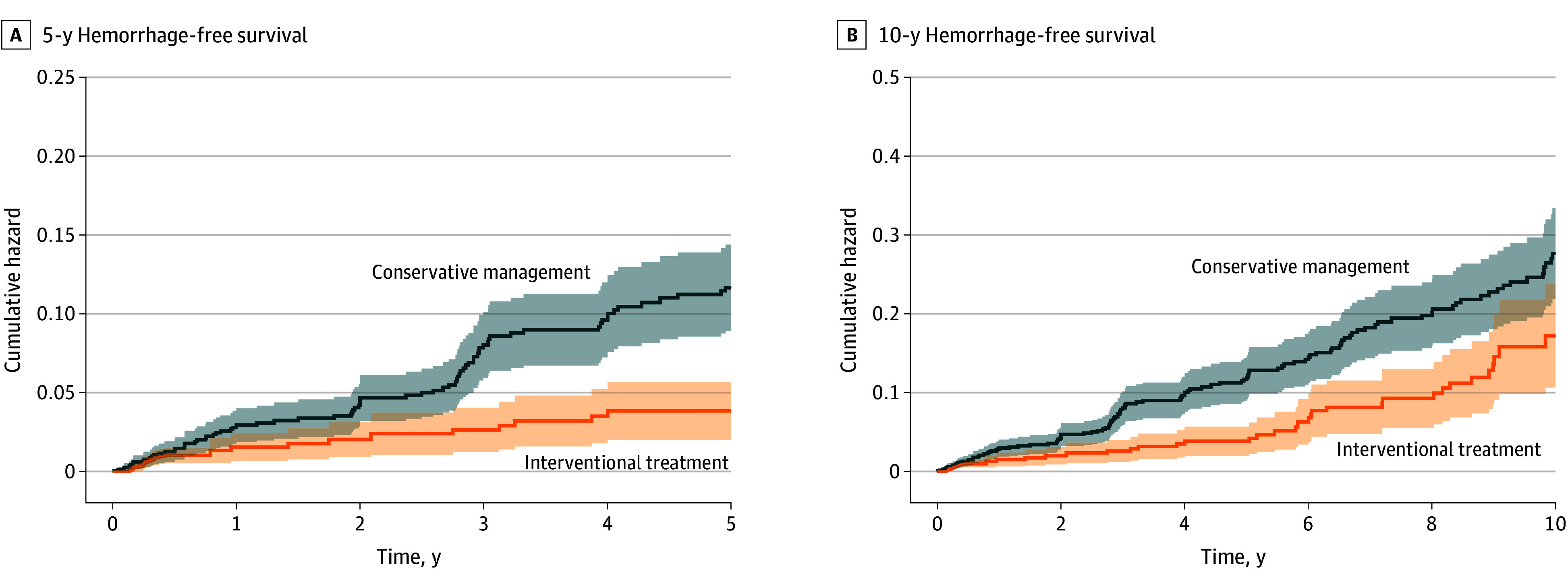
Weighted Cumulative Hazard Curves Comparing Treatment Strategies in the Emulated Trial Using the Cloned Cohort

After cloning, by 5 years of follow-up, 78 hemorrhages occurred in the conservative management group and 27 occurred in the interventional treatment group. The annual incidence of hemorrhage was 2.44% in the conservative management group and 1.47% in the interventional treatment group at 5 years and 2.62% and 1.75%, respectively, at 10 years. In the CCW analysis, intervention was associated with a significantly higher probability of remaining hemorrhage free. At 5 years, the estimated hemorrhage-free survival was 96.23% (95% CI, 93.95%-97.65%) for the interventional treatment group vs 89.00% (95% CI, 86.37%-91.24%) for the conservative management group (Table 2), an absolute difference of 7.23% (95% CI, 4.78%-9.91%). The weighted HR for hemorrhage with interventional treatment vs conservative management was 0.44 (95% CI, 0.33-0.57), indicating a substantially lower hazard of hemorrhage with early intervention. At 10 years, cumulative incidence of hemorrhage remained lower among patients receiving intervention, with an absolute difference of 8.37% (95% CI, 2.68%-15.70%) and weighted HR of 0.56 (95% CI, 0.42-0.69). These findings are illustrated by IPCW-weighted survival curves (Figure 2). The unweighted curves and those based on receipt of actual treatment showed similar results (eFigures 4 and 5 in Supplement 1).

**Table 2.** Patient Survival Outcomes 5 and 10 Years After Diagnosis

Variable	5-y Follow-up		10-y Follow-up	
	Conservative management (n = 1264)	Interventional treatment (n = 539)	Conservative management (n = 1264)	Interventional treatment (n = 539)
Hemorrhagic stroke or death, No. (%)	78 (6.17)	27 (5.01)	120 (9.49)	47 (8.72)
Annual incidence rate, %	2.44	1.47	2.62	1.75
Probability of hemorrhage-free survival, %	89.00 (86.37-91.24)	96.23 (93.95-97.65)	75.84 (71.28-79.99)	84.21 (77.63-88.88)
Difference in survival, % (95% CI)^a^	0 [Reference]	7.23 (4.78-9.91)	0 [Reference]	8.37 (2.68-15.70)
Hazard ratio (95% CI)^a^	1 [Reference]	0.44 (0.33-0.57)	1 [Reference]	0.56 (0.42-0.69)

^a^ Estimands were calculated with the conservative management group set as the reference. The 95% CIs are derived from nonparametric bootstrapping with 200 replicates.

### Subgroup and Sensitivity Analyses

**Figure 3.**
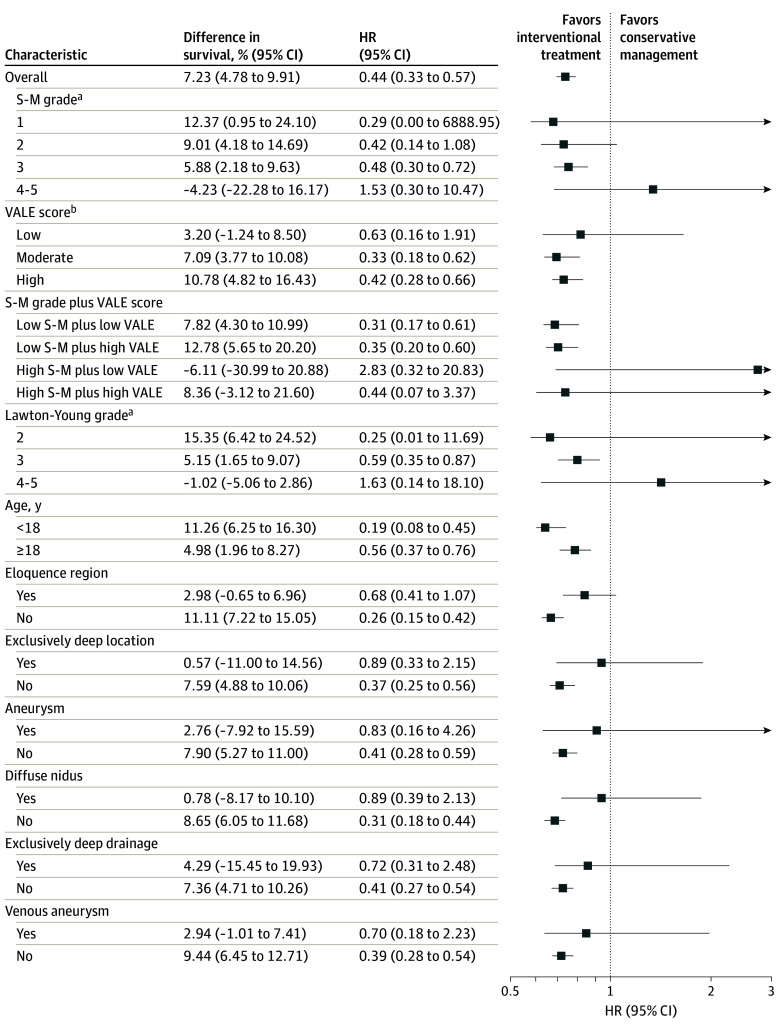
Subgroup Analyses for 5-Year Hemorrhage-Free Survival HR indicates hazard ratio; S-M, Spetzler-Martin; and VALE, ventricular system involvement, venous aneurysm, deep location, and exclusively deep drainage. ^a^Higher grades indicate greater surgical complexity and risk. ^b^Higher scores indicate higher risk.

Figure 3 presents the results of prespecified subgroup analyses for the 5-year outcome. The benefit of intervention for hemorrhage-free survival and lower hemorrhage hazard was generally consistent across most subgroups. However, in certain high-risk subgroups, such as patients with high-grade AVMs (S-M grades 4-5), outcomes favored conservative management. In this subgroup, the HR was 1.53 (95% CI, 0.30-10.47), with a difference in hemorrhage-free survival of −4.23% (95% CI, −22.28% to 16.17%), suggesting potentially higher hemorrhage risk with intervention, although 95% CIs were wide. A similar pattern was observed for patients with high S-M grade and low VALE score, indicating complex but low-risk lesions. In this subgroup, the 5-year outcome also favored conservative management (HR, 2.83 [95% CI, 0.32-20.83]; survival difference, −6.11% [95% CI, −30.99% to 20.88%]). For most other subgroups, including those defined by angiographic features, intervention was associated with equal or better outcomes. These findings were consistent at 10 years (eFigure 6 in Supplement 1). However, the diffuse nidus subgroup showed a time-dependent reversal: while intervention initially conferred benefit, by 10 years hemorrhage risk had increased, but the increase was not significant (HR, 1.15 [95% CI, 0.73-1.81]; survival difference, −7.78% [95% CI, −21.36% to 5.32%]).

When modalities were analyzed separately, only microsurgical resection remained associated with reduced hemorrhage risk across all outcomes. Modifying the grace period for treatment initiation, adjusting eligibility criteria, conducting complete-case analysis, using the propensity score method, or incorporating functional status into the outcomes did not substantially alter the observed probable treatment effect (eTable 6 in Supplement 1).

### Exploratory Nested Case-Control Analysis

In the nested case-control study, 105 patients with incident hemorrhage were each matched to up to 4 controls sampled from their respective risk sets. In the multivariable analysis, interventional treatment and vein aneurysm remained independently associated with lower hemorrhage risk, whereas ventricular system involvement and venous stenosis were independently associated with increased risk (eTable 7 in Supplement 1).

## Discussion

In this study, we evaluated the association of interventional treatment vs conservative management with hemorrhage-free survival in a cohort of patients with unruptured AVMs. By applying the CCW method and IPCW, we found a significantly lower 5- to 10-year risk of intracranial hemorrhage among patients who underwent intervention. Subgroup analyses indicated this protective effect was generally consistent, although certain high-risk subsets, such as patients with high S-M grades or complex anatomy, derived less benefit.

The estimated long-term protective effect we observed contrasts with the findings from ARUBA, which reported markedly worse outcomes in the interventional treatment group after a mean (SD) follow-up of 33.3 (19.7) months.^5^ While the annual hemorrhage rate of 2.6% in ARUBA’s conservative management group was similar to our estimates (2.44% at 5 years and 2.62% at 10 years) and to those reported by Hernesniemi et al^24^ (2.4% during the decades-long follow-up), the interventional treatment group results were poorer than those reported in virtually all other interventional series.^7,9,11^ In ARUBA, nearly one-third of patients in the interventional treatment group experienced stroke or death (30.7%), equating to an annual hemorrhage rate of 10.6%. By contrast, in our study, the annual rate among treated patients was 1.47%, and most other large studies also reported considerably lower risks. This discrepancy reflects that ARUBA was an outlier in terms of how unfavorable the intervention results were, as confirmed by bayesian reanalyses,^6^ likely because of its trial setup, with short follow-up, heterogeneous and often noncurative treatment strategies, and less-than-optimal delivery of interventions.^12,25^ Therefore, the results of ARUBA should be interpreted with caution: the trial provides important randomized evidence, but its design and execution led to intervention outcomes that were not representative of contemporary practice. Ultimately, trial findings are only as reliable as the quality and completeness of the interventions being tested, and achieving this standard in unruptured AVMs remains challenging, given the low event rates and long follow-up required.

In this context, target trial emulation offers a feasible alternative to approximate the evidence that a rigorous trial might have provided using clinical data.^17,26^ By explicitly defining a hypothetical trial, we emulated this idealized design using comprehensive registry data. This approach enabled us to mitigate biases commonly encountered in observational studies, such as immortal time bias and baseline confounding.^27^ Prior research has shown that well-executed emulations can closely match actual randomized clinical trials.^28^ Therefore, by applying CCW within this framework, we derived meaningful causal inferences from clinical data. Beyond AVMs, this approach may guide research in similarly rare conditions with infrequent but serious outcomes.^16^

Our subgroup analyses showed that intervention benefit is not uniform and depends on lesion characteristics. High S-M grade AVMs, for instance, did not experience the same degree of benefit, aligning with long-standing clinical judgment of high operative risk.^29^ Similarly, patients with both high S-M grade and low VALE score (indicating a lesion that is difficult to treat but with a low risk for rupture), fared no better with intervention, and possibly worse. Numerous AVM grading systems exist beyond S-M and VALE, each designed to capture different aspects of treatment risk and complexity. For example, the Buffalo score^30^ and AVM Embocure score^31^ are tailored to predict outcomes following endovascular treatment, while the Virginia Radiosurgery AVM Scale was developed for SRS.^32^ These divergent findings imply a need for individualized treatment planning, ideally guided by multiple grading systems, rather than any single framework.

An interesting finding emerged in patients with diffuse nidus AVMs: intervention was initially protective at 5 years, but by 10 years the outcomes had reversed. This likely reflects the difficulty of achieving complete obliteration in diffuse lesions. Initial treatment may reduce shunt flow and offer temporary benefit, but residual nidus or altered hemodynamics can predispose to later hemorrhage.^33,34^ Our findings suggest that early success in treating diffuse AVMs does not guarantee durable protection, and long-term surveillance is necessary. In such cases, the decision between intervention and observation requires balancing short-term benefits against late complications. This also reinforces the need for extended follow-up in complex AVMs.

Another important aspect of our analysis is the breakdown of outcomes by treatment modality. Neither endovascular embolization alone nor SRS alone showed a significant advantage in isolation in the sensitivity analyses. This result is consistent with known differences in the efficacy of nidus obliteration across modalities.^35^ Microsurgery, when successful, achieves immediate and complete removal of the nidus, effectively eliminating future hemorrhage risk.^13^ In contrast, embolization is often adjunctive or partial; complete cure by embolization alone is uncommon except in select cases, and most patients retain residual nidus tissue with ongoing rupture risk.^36^ Similarly, SRS involves a latency period of 2 to 3 years before thrombosis occurs, when patients remain at risk, and even afterward complete obliteration is not guaranteed, particularly for larger or diffuse AVMs.^37^ These findings suggest that the benefit of intervention observed in our primary analysis was largely driven by surgical resection. However, this should not be interpreted as a blanket recommendation for surgery. Type and quality of intervention are critical, and optimal treatment must be tailored to the individual lesion and clinical context. It is also important to note that radiosurgical and endovascular strategies frequently necessitate repeated sessions or combination with other modalities, each of which carries additional risk. The cumulative risks of multiple treatment warrant further investigation in larger datasets with longer follow-up.

### Limitations

Several limitations should be considered. First, our primary outcome was limited to hemorrhage, and we did not assess other important clinical end points such as neurologic deficits, seizures, or cognitive function. Similarly, treatment-specific outcomes, including obliteration rates, ischemic stroke, or adverse radiation effects, were not analyzed because they are not directly comparable between interventional treatment and conservative management. Second, data were drawn from high-volume tertiary centers in the MATCH registry, where specialized expertise and advanced technology likely reduced the rate of hemorrhage. Therefore, the event rates observed in the interventional treatment group may underestimate those achievable in less specialized settings. Third, despite rigorous methods to simulate randomization, residual confounding may persist. While known covariates were accounted for, unmeasured factors could still influence treatment selection and outcomes. In addition, the follow-up schedule in the cohort study may have introduced bias in event recording. Finally, subgroup and sensitivity analyses should be interpreted cautiously, as increased stratification reduces sample sizes and can introduce statistical instability.

## Conclusions

In this emulated target trial comparing interventional treatment and conservative management in a cohort of patients with unruptured AVMs, we found a significant reduction in hemorrhage risk associated with intervention during 5- and 10-year follow-up periods. However, these benefits were not uniformly observed across all lesion types and intervention modalities. Given the inherent challenges of randomized clinical trials in rare conditions, our methodology offers a robust approach to evaluating clinical strategies in settings where definitive randomized evidence remains elusive.

## Notes

Supplement 1. **eMethods 1.** Protocol for Data Quality Management **eMethods 2.** Inverse Probability-of-Censoring Weighting (IPCW) Procedure **eMethods 3.** Measures for Reducing Follow-Up Bias **eMethods 4.** Description of the Exploratory Nested Case-Control Design **eMethods 5.** Description of the Sensitivity Analyses **eFigure 1.** Visual depiction of the Treatment Patterns **eFigure 2.** Visual Depiction of the Censoring Rules for Primary Analysis **eFigure 3.** Standardized Mean Differences at the End of the 6-Month Grace Period **eFigure 4.** Unweighted Cumulative Hazard Curves Comparing Treatment Strategies in the Emulated Trial Using the Cloned Cohort **eFigure 5.** Unweighted Cumulative Hazard Curves Comparing Treatment Strategies According to Receipt of Actual Treatment **eFigure 6.** Subgroup Analyses for 10-Year Hemorrhage-Free Survival **eTable 1.** Protocol of the Hypothetical Target Trial and Emulation **eTable 2.** Breakdown of Missing Data According to Receipt of Actual Treatment **eTable 3.** Baseline Demographic and AVM Characteristics According to Receipt of Actual Treatment **eTable 4.** Detailed Treatment Distribution and Follow-Up in the Intervention and Conservative Management Groups **eTable 5.** Baseline Characteristics of Patients Treated With and Without Microsurgery in the Intervention Group **eTable 6.** Results for Sensitivity Analyses **eTable 7.** Results of the Nested Case-Control Analysis for Hemorrhage Predictive Factors

Supplement 2.
Nonauthor Collaborators

Supplement 3.
Data Sharing Statement
