# Supplementary material for: Interventional Treatment vs Conservative Management of Unruptured Brain Arteriovenous Malformations
Source: JAMA Netw Open. 2025 Nov 13;8(11):e2543408. doi: 10.1001/jamanetworkopen.2025.43408 (PMC12616460; doi:10.1001/jamanetworkopen.2025.43408)
Supplement: Supplement 1. — eMethods 1. Protocol for Data Quality Management eMethods 2. Inverse Probability-of-Censoring Weighting (IPCW) Procedure eMethods 3. Measures for Reducing Follow-Up Bias eMethods 4. Description of the Exploratory Nested Case-Control Design eMethods 5. Description of the Sensitivity Analyses eFigure 1. Visual depiction of the Treatment Patterns eFigure 2. Visual Depiction of the Censoring Rules for Primary Analysis eFigure 3. Standardized Mean Differences at the End of the 6-Month Grace Period eFigure 4. Unweighted Cumulative Hazard Curves Comparing Treatment Strategies in the Emulated Trial Using the Cloned Cohort eFigure 5. Unweighted Cumulative Hazard Curves Comparing Treatment Strategies According to Receipt of Actual Treatment eFigure 6. Subgroup Analyses for 10-Year Hemorrhage-Free Survival eTable 1. Protocol of the Hypothetical Target Trial and Emulation eTable 2. Breakdown of Missing Data According to Receipt of Actual Treatment eTable 3. Baseline Demographic and AVM Characteristics According to Receipt of Actual Treatment eTable 4. Detailed Treatment Distribution and Follow-Up in the Intervention and Conservative Management Groups eTable 5. Baseline Characteristics of Patients Treated With and Without Microsurgery in the Intervention Group eTable 6. Results for Sensitivity Analyses eTable 7. Results of the Nested Case-Control Analysis for Hemorrhage Predictive Factors [file jamanetwopen-e2543408-s001.pdf]

Supplementary Online Content

Han H, Chen Y, Ma L, et al; Registry of Multimodality Treatment for Brain Arteriovenous Malformation in Mainland China (MATCH). Interventional treatment vs conservative management of unruptured brain arteriovenous malformations. *JAMA Netw Open.* 2025;8(11):e2543408. doi:10.1001/jamanetworkopen.2025.43408

eMethods 1. Protocol for Data Quality Management

eMethods 2. Inverse Probability-of-Censoring Weighting (IPCW) Procedure

eMethods 3. Measures for Reducing Follow-Up Bias

eMethods 4. Description of the Exploratory Nested Case-Control Design

eMethods 5. Description of the Sensitivity Analyses

eFigure 1. Visual depiction of the Treatment Patterns

eFigure 2. Visual Depiction of the Censoring Rules for Primary Analysis

eFigure 3. Standardized Mean Differences at the End of the 6-Month Grace Period

eFigure 4. Unweighted Cumulative Hazard Curves Comparing Treatment Strategies in the Emulated Trial Using the Cloned Cohort

eFigure 5. Unweighted Cumulative Hazard Curves Comparing Treatment Strategies According to Receipt of Actual Treatment

eFigure 6. Subgroup Analyses for 10-Year Hemorrhage-Free Survival

eTable 1. Protocol of the Hypothetical Target Trial and Emulation

eTable 2. Breakdown of Missing Data According to Receipt of Actual Treatment

eTable 3. Baseline Demographic and AVM Characteristics According to Receipt of Actual Treatment

eTable 4. Detailed Treatment Distribution and Follow-Up in the Intervention and Conservative Management Groups

eTable 5. Baseline Characteristics of Patients Treated With and Without Microsurgery in the Intervention Group

eTable 6. Results for Sensitivity Analyses

eTable 7. Results of the Nested Case-Control Analysis for Hemorrhage Predictive Factors

This supplementary material has been provided by the authors to give readers additional information about their work.

© 2025 Han H et al. *JAMA Network Open*.

eMethods 1. Protocol for Data Quality Management

1. Definition of variables were discussed and unified according to the terminology reporting standards or published paper before the initiation of data collection. Clinical research coordinators (CRCs) and neurosurgery residents were then trained by cerebrovascular neurosurgeons with more than 15 years' working experience. CRCs were responsible for demographic information and follow-up data, and neurosurgery residents for angiographic features. The two parts were blinded to each other to ensure the data collected were not biased by imaging characteristics or clinical outcomes.
2. A standard training dataset with 50 cases were used to check the consistency of data collectors. For those variables or cases with significant interobserver variation, the consensus was reached by either modifying the confusing definitions or retraining the data collectors. Only when the consistency reached 90% can the CRC or the resident allowed to extracting information independently.
3. While recording data, one could ask for help about unsure cases in a discussion group with cerebrovascular neurosurgeons in it, or mark these cases and discuss in weekly meetings.
4. The group leader with more than five years' working experience randomly spot checks these data biweekly. Investigators would receive training again if their data were of low quality, and these data would be recollected by other investigators.

eMethods 2. Inverse Probability-of-Censoring Weighting (IPCW) Procedure

The clone-censor-weight (CCW) design creates two virtually randomized arms at baseline but introduces informative censoring over time because treatment decisions are related to patient prognostic factors. We addressed this potential selection bias by applying IPCW. IPCW up-weights uncensored observations to represent those that were censored with similar covariate profiles, thereby preserving comparability between arms and enabling unbiased estimation of the causal effect.

Because the censoring process was influenced by patient characteristics, we modeled the probability of remaining uncensored in each arm and used those models to derive IPC weights. Separate censoring models were fit for the intervention and control arms, reflecting their different censoring mechanisms. Both models adjusted for the full set of baseline covariates listed in the Methods section (including demographic, clinical, and radiographic features) to account for confounding.

Intervention arm (Tx): For the main analysis, the only opportunity for deviation in the Tx arm was at the end of the grace period. If a Tx-assigned patient had not initiated the intervention by 6 months, that clone was classified as non-adherent to the immediate-treatment strategy and was censored at 6 months (the point of protocol deviation). We estimated a time-fixed IPC weight for each Tx clone based on the probability of adhering to the assigned treatment by 6 months. A multivariable logistic regression was used to model the odds of initiating treatment within 6 months (versus not initiating by 6 months) as a function of baseline covariates. From this model, we obtained each patient's predicted probability of remaining uncensored through 6 months. Tx clones that adhered were assigned an IPC weight equal to the inverse of this probability. Clones censored at 6 months (for no treatment) did not contribute further follow-up, but their information was accounted for by the weights on uncensored clones. Because adherence could only be determined at a single time point for Tx clones, these weights were time-fixed throughout the remaining follow-up.

Control arm: If a control-assigned patient underwent an interventional treatment at any time during follow-up (i.e. crossover to the intervention), their control clone was censored at the time of treatment. Thus, control clones could be censored at various time points whenever a crossover occurred. We fit a multivariable Cox model for the time to censoring (time to first intervention) in the control arm. This arm-specific Cox model included all baseline covariates and produced an estimated hazard for protocol deviation at each time. From the Cox model, we computed the survival probability of each control clone remaining uncensored up to each point in time. Implementation involved a risk-set approach with the data split at each observed censoring event time and calculation of each clone's probability of not yet being censored at that time, given their covariates. The inverse of this conditional probability was used as the weight contribution for that time interval. As follow-up progressed, each control clone's weight was updated over time: at each censoring event, clones still at risk had their weight multiplied by the inverse of their predicted probability of remaining uncensored through that interval. The detailed codes and mechanisms for implementing the multivariable time-dependent model referred to Willems's work (*Willems S, Schat A, van Noorden MS, Fiocco M. Correcting for dependent censoring in routine outcome monitoring data by applying the inverse probability censoring weighted estimator. Stat Methods Med Res. 2018 Feb;27(2):323-335. doi: 10.1177/0962280216628900. Epub 2016 Mar 17*).

Extreme IPC weights were truncated to avoid excessive variance and prevent undue influence of observations with extremely low probabilities of remaining uncensored. In sensitivity analyses, when outcomes were separately analyzed for each single treatment modality (e.g., SRS vs. conservative

© 2025 Han H et al. *JAMA Network Open*.

management), censoring dates were no longer time-fixed, as patients assigned to a specific modality who either did not initiate treatment within 6 months or received a different modality were censored accordingly. Thus, time-varying IPC weighting methods used in the control arm were applied to estimate weights in these scenarios. Additionally, in subgroup analyses, covariates that exhibited no variability (having only a single value within the subgroup) were excluded from the multivariable models to avoid violating the positivity assumption necessary for valid IPCW implementation.

© 2025 Han H et al. *JAMA Network Open*.

eMethods 3. Measures for Reducing Follow-Up Bias

1. A follow-up schedule is established at the beginning of the study. Patients are informed of regular follow-up visits at 3 months, annually (1, 2, and 3 years), and every 5 years after the treatment.
2. To increase participant retention, we aim to build a rapport with participants from the outset. We build online chatgroups for the convenience of doctor-patient and patient-patient communication. In this online community, we explain the purpose and importance of the study, answer their questions, and provide regular updates about the study's progress. We also provide feedback on their contribution to the study and share relevant results (where appropriate). A telemedicine applet (WeChat) is also used for online consultation and follow-up.
3. In cases where participants do not respond to follow-up attempts, we implement a standardized protocol for additional contact attempts. This involves using alternate contact methods or reaching out to outpatient medical records. If these attempts turned out to be unsuccessful, the participant is classified as lost to follow-up.
4. Despite our best efforts, some participants may be lost to follow-up. We regularly compare characteristics in patients included and lost to follow-up according to different study designs. Similar characteristics would imply the rationale for analyzing the included data with relatively low follow-up bias.
5. Similar to data collection, the follow-up process is reviewed periodically by the group leader. Any issues or areas for improvement are identified and addressed. Refresher training sessions are conducted as needed to ensure that clinical research coordinator members are up-to-date on the follow-up protocol and best practices.

© 2025 Han H et al. *JAMA Network Open*.

eMethods 4. Description of the Exploratory Nested Case-Control Design

We conducted a risk set-based nested case-control (NCC) analysis within the emulated cohort. After cloning individuals to create intervention and conservative-management arms, we combined the 2 arms into a single analytic file. For each individual, follow-up time (years) was measured from the index date to the earliest of intracranial hemorrhage, treatment censoring as defined in the emulation, or administrative end of follow-up.

Cases were all individuals who experienced a first hemorrhage during follow-up. For each case, we formed a risk set comprising all cohort members who were event free and still under observation at the case's event time using incidence-density sampling. From each risk set we selected up to 4 controls by simple random sampling with equal probability. Within a given risk set, the same individual was not sampled more than once; across different risk sets, the same control could be sampled again if still at risk at later event times. Each case-control set received a unique stratum identifier used in the conditional likelihood.

We fitted conditional logistic regression models using an exact conditional likelihood with strata defined by the case-specific risk sets. Candidate predictors were evaluated as recorded at baseline and included sex, age, presentation variables, angiographic features, and treatment arm. To avoid collinearity, composite grading scales were excluded from the regression analysis, as their component features were directly modeled as candidate predictors. Univariable conditional logistic regressions were first performed for each candidate predictor; variables with $P < 0.05$ were then included in the multivariable model to identify independent predictors of hemorrhage.

© 2025 Han H et al. *JAMA Network Open*.

eMethods 5. Description of the Sensitivity Analyses

Several sensitivity analyses were performed to test the robustness of our findings.

1. **Alteration of the grace period**

In the main analysis, patients assigned to the intervention arm were allowed a 6-month grace period after diagnosis to initiate treatment. To test whether this choice affected the results, we repeated the analysis using shorter (3 months) and longer (12 months) grace periods. Patients who did not initiate the assigned treatment within the specified grace period were censored at that time.

2. **Treatment modality-specific analyses**

Each treatment modality harbors different efficacy profiles and risks. To disentangle their contributions, we reported single-modality sensitivity analyses. We evaluated outcomes separately for each intervention modality (microsurgery, stereotactic radiosurgery [SRS], or embolization) compared with conservative management. Patients were censored at the time of treatment switching, receipt of an alternative modality, or lack of initiation of the assigned modality within 6 months. Follow-up ended at the earliest occurrence of outcome event, treatment switching, last contact, or 5/10 years.

3. **Restricted cohort of ARUBA-eligible patients**

To facilitate comparison with the ARUBA trial, we repeated the primary analysis in a subset of patients who met ARUBA eligibility criteria.

4. **Complete-case analysis**

Because our primary analysis used the imputed dataset, we conducted a sensitivity analysis restricted to patients with complete baseline data.

5. **Propensity score-matching (PSM) analysis**

As an alternative strategy to adjust for confounding, we applied PSM method. Propensity scores for receiving intervention were estimated using baseline demographic, clinical, and radiographic covariates. Patients were originally grouped according to receipt of actual treatment, and were matched 1:1 between intervention and conservative groups using nearest-neighbor matching without replacement, with a caliper radius of 0.2. Survival analyses were then repeated within the matched cohort.

6. **Redefinition of the outcome**

Because prevention of hemorrhage alone does not capture the full clinical impact of treatment, we broadened the definition of the endpoint to include functional status. In this analysis, the outcome was defined as hemorrhage resulting in a modified Rankin Scale ≥2.

© 2025 Han H et al. *JAMA Network Open*.

eFigure 1. Visual depiction of the Treatment Patterns

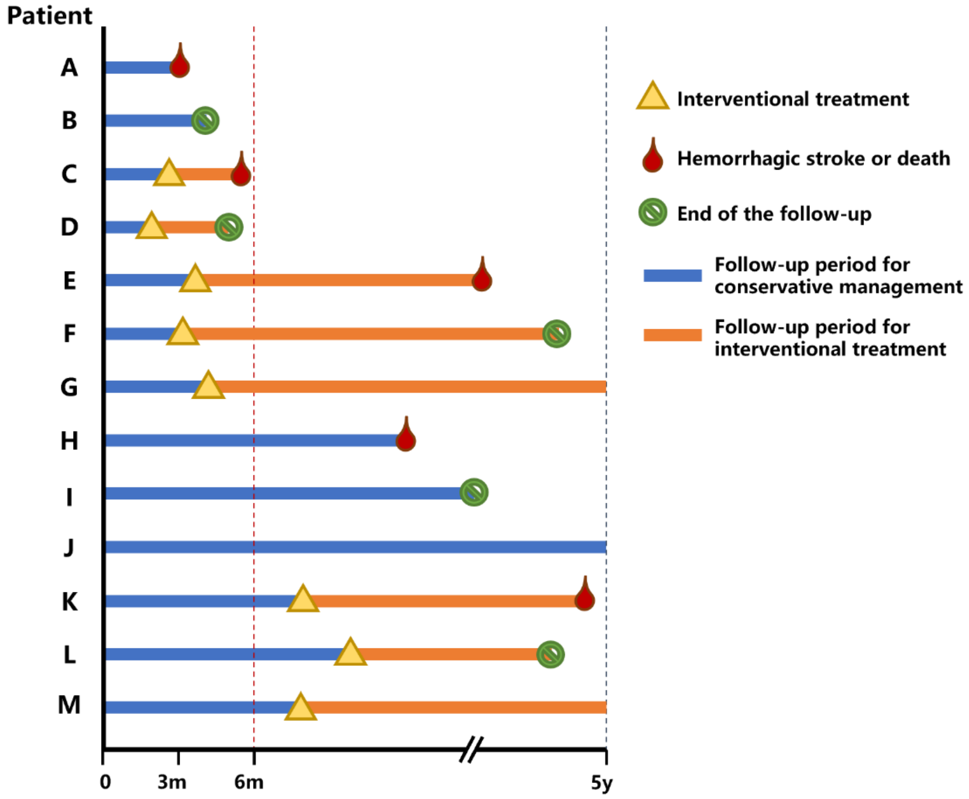

In traditional observational study designs, patients are categorized based on their eventual treatment decisions (e.g., Patients A, B, H, I, and J classified as receiving conservative management, and the remainder as receiving intervention), which may introduce immortal time bias due to differential timing of treatment initiation. In contrast, our target trial emulation applies a clone-censor-weight approach, whereby each patient is duplicated and assigned to both treatment strategies. Each clone is then followed under their assigned strategy, with artificial censoring applied at the point of deviation from the assigned treatment protocol. The censoring rules are visually illustrated in eFigure 2. For patients receiving interventional treatment, specific procedural strategies were evaluated in sensitivity analyses, and follow-up was censored at the earliest of: the first hemorrhagic event, the last available follow-up, or the administrative end of follow-up (5 years in this illustration).

© 2025 Han H et al. *JAMA Network Open*.

eFigure 2. Visual Depiction of the Censoring Rules for Primary Analysis

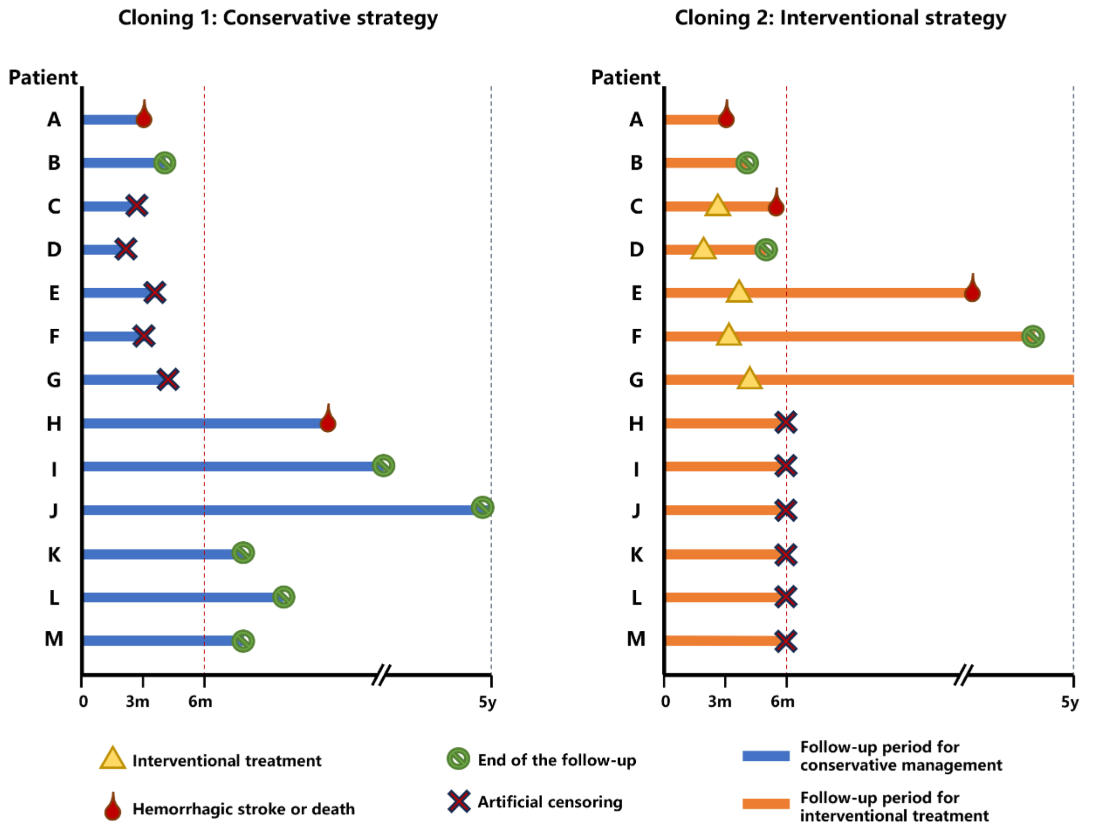

In the clone-censor-weight framework, patient data are duplicated, and each clone is assigned to follow either strategy 1 (no interventional treatment within 6 months) or strategy 2 (receipt of interventional treatment within 6 months). Censoring status is determined based on adherence to the assigned strategy. To account for the selection bias introduced by artificial censoring, two models are employed: a censoring model to estimate inverse-probability-of-censoring weights, and a weighted outcome model to estimate the causal effect.

Patients A and B contribute equally to both arms, with censoring indicators set to 0, as they do not deviate from either strategy. Their follow-up ends due to either the outcome of interest or administrative censoring before any treatment decision can be assessed. Patients C–G undergo interventional treatment within the grace period and are therefore considered adherent in the intervention arm (censoring indicator = 0), but non-adherent in the control arm (censoring indicator = 1). These patients contribute to the censoring model in both arms until the time of intervention, and in the analysis model, they are censored at their treatment time in the control arm. Patients H–M comply with the conservative strategy throughout the 6-month grace period. Accordingly, they are not censored in the control arm (censoring indicator = 0) but are artificially censored at 6 months in the intervention arm for deviating from that strategy (censoring indicator = 1). These patients contribute the full grace period to the censoring model, and are censored at 6 months in the intervention arm analysis.

As illustrated in the cloning diagram, censoring for the intervention arm occurs at a fixed time point (6 months), and therefore time-fixed IPC weights are applied for clones in this arm (as described in eMethod3). In sensitivity analyses examining specific interventional modalities, such as radiosurgery alone, patients C–G may be artificially censored depending on their assigned strategy arm and actual treatment received.

© 2025 Han H et al. *JAMA Network Open*.

eFigure 3. Standardized Mean Differences at the End of the 6-Month Grace Period

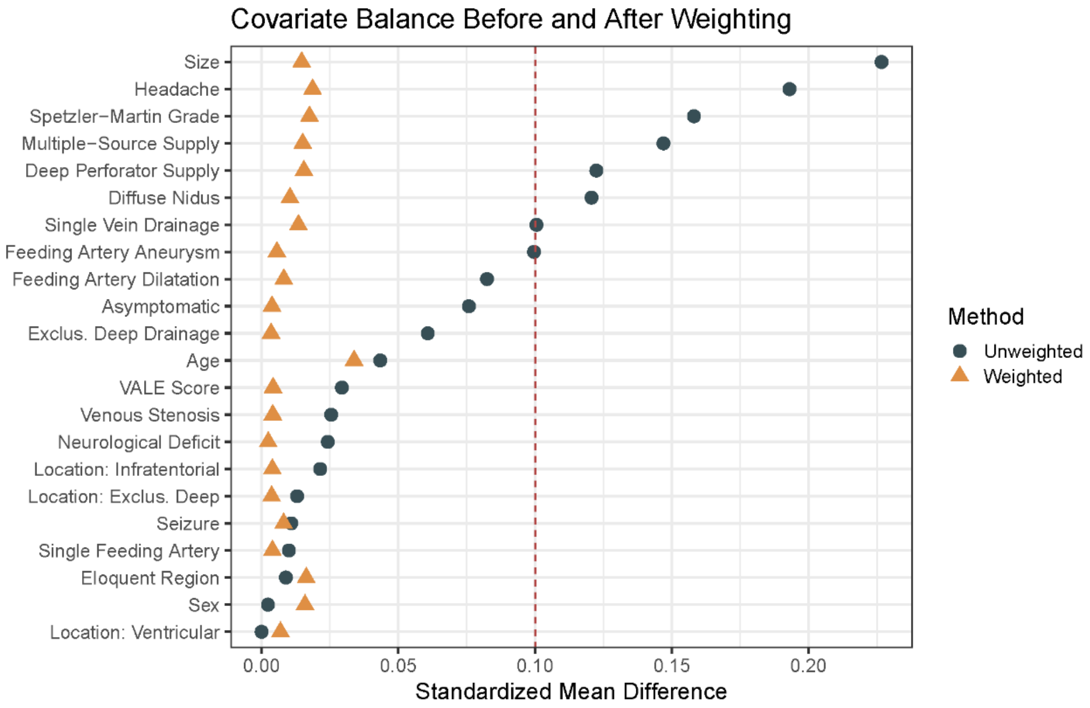

© 2025 Han H et al. *JAMA Network Open*.

eFigure 4. Unweighted Cumulative Hazard Curves Comparing Treatment Strategies in the Emulated Trial Using the Cloned Cohort

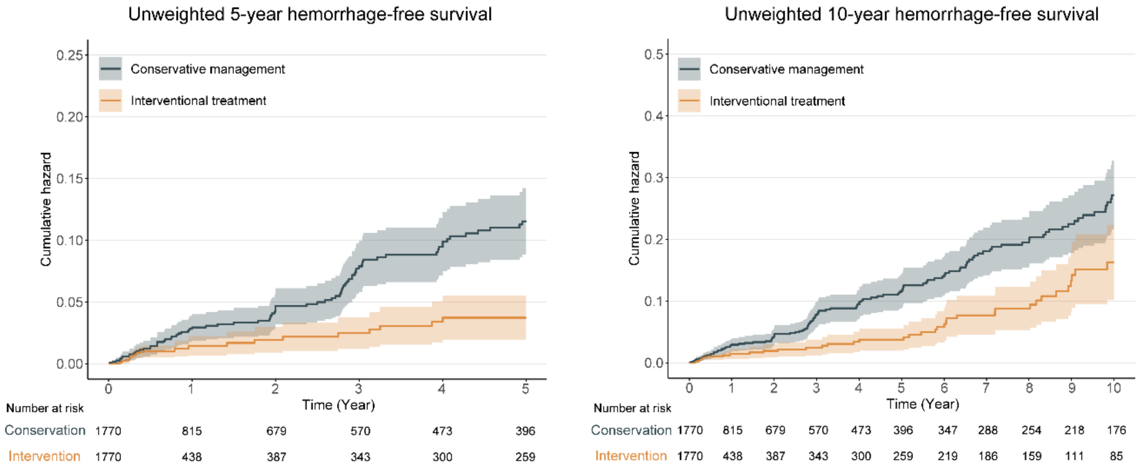

© 2025 Han H et al. *JAMA Network Open*.

eFigure 5. Unweighted Cumulative Hazard Curves Comparing Treatment Strategies According to Receipt of Actual Treatment

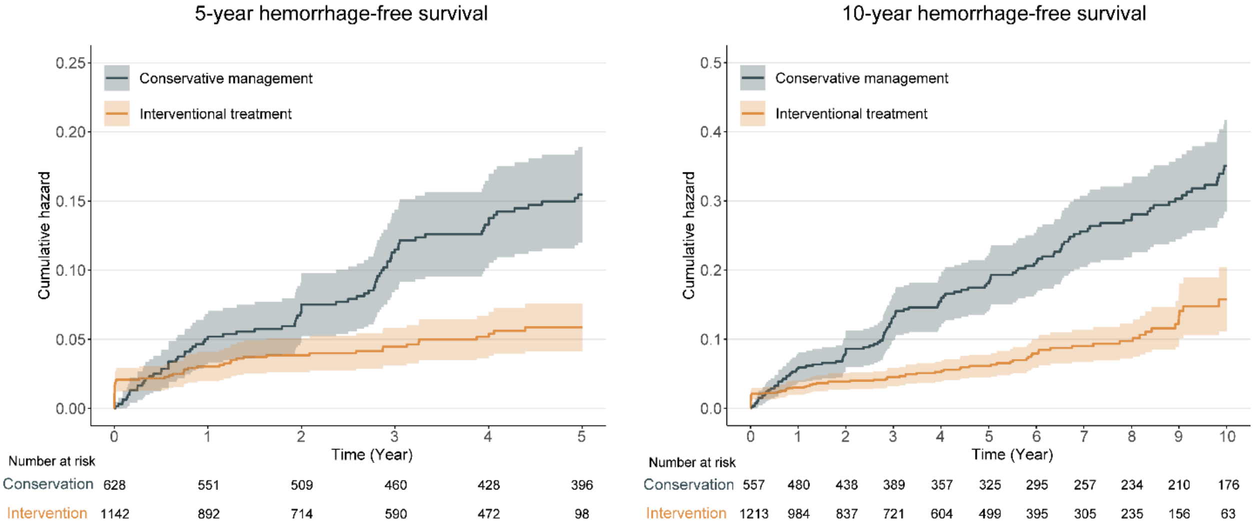

In this analysis, patients are grouped by the treatment they ultimately received rather than by the emulated assignment. For the interventional group, time zero is defined as the date of treatment initiation, in contrast to the target trial emulation design where time zero is set at the date of AVM diagnosis.

© 2025 Han H et al. *JAMA Network Open*.

eFigure 6. Subgroup Analyses for 10-Year Hemorrhage-Free Survival

Characteristics	Difference in survival (95% CI)	HR (95%CI)
Overall	8.36% (2.68–15.70%)	0.56 (0.42–0.69)
**S–M grade**		
1	6.79% (–10.61–25.33%)	0.37 (0.01–19.78)
2	12.39% (3.88–22.53%)	0.51 (0.23–0.89)
3	7.50% (–2.46–19.07%)	0.60 (0.39–0.88)
4~5	–11.73% (–29.80–8.94%)	1.54 (0.64–3.63)
**VALE score**		
Low	9.45% (–2.44–19.74%)	0.44 (0.09–1.85)
Moderate	9.45% (1.94–19.11%)	0.42 (0.24–0.64)
High	4.95% (–7.63–16.09%)	0.68 (0.49–1.00)
**S–M+VALE**		
Low SM+Low VALE	13.63% (5.41–20.13%)	0.30 (0.18–0.55)
Low SM+High VALE	0.35% (–14.33–16.59%)	0.69 (0.44–1.09)
High SM+Low VALE	–19.73% (–52.77–13.57%)	2.66 (0.54–14.97)
High SM+High VALE	15.68% (–6.02–47.47%)	0.61 (0.24–1.30)
**L–Y grade**		
2	20.10% (–2.15–34.24%)	0.49 (0.28–0.81)
3	7.05% (–2.29–15.98%)	0.64 (0.43–0.92)
4~5	–3.54% (–10.31–5.19%)	1.53 (0.64–2.91)
**Age**		
< 18	6.31% (–2.98–18.24%)	0.53 (0.31–0.79)
≥ 18	8.83% (2.18–15.61%)	0.59 (0.42–0.78)
**Eloquence region**		
Yes	2.52% (–6.27–12.10%)	0.79 (0.53–1.10)
No	11.82% (2.37–19.90%)	0.38 (0.24–0.64)
**Exclusively deep location**		
Yes	4.48% (–15.06–21.99%)	0.82 (0.37–1.93)
No	7.78% (1.19–14.18%)	0.53 (0.39–0.71)
**Aneurysm**		
Yes	3.44% (–18.79–26.43%)	0.88 (0.34–1.82)
No	8.47% (2.60–13.93%)	0.53 (0.40–0.68)
**Diffuse nidus**		
Yes	–7.78% (–21.36–5.32%)	1.15 (0.73–1.81)
No	15.64% (9.96–22.94%)	0.35 (0.23–0.47)
**Exclusively deep drainage**		
Yes	12.03% (–12.12–34.64%)	0.69 (0.33–1.60)
No	8.13% (1.90–14.42%)	0.53 (0.37–0.71)
**Venous aneurysm**		
Yes	15.24% (7.38–23.78%)	0.39 (0.14–0.95)
No	6.28% (–1.90–13.05%)	0.57 (0.44–0.77)

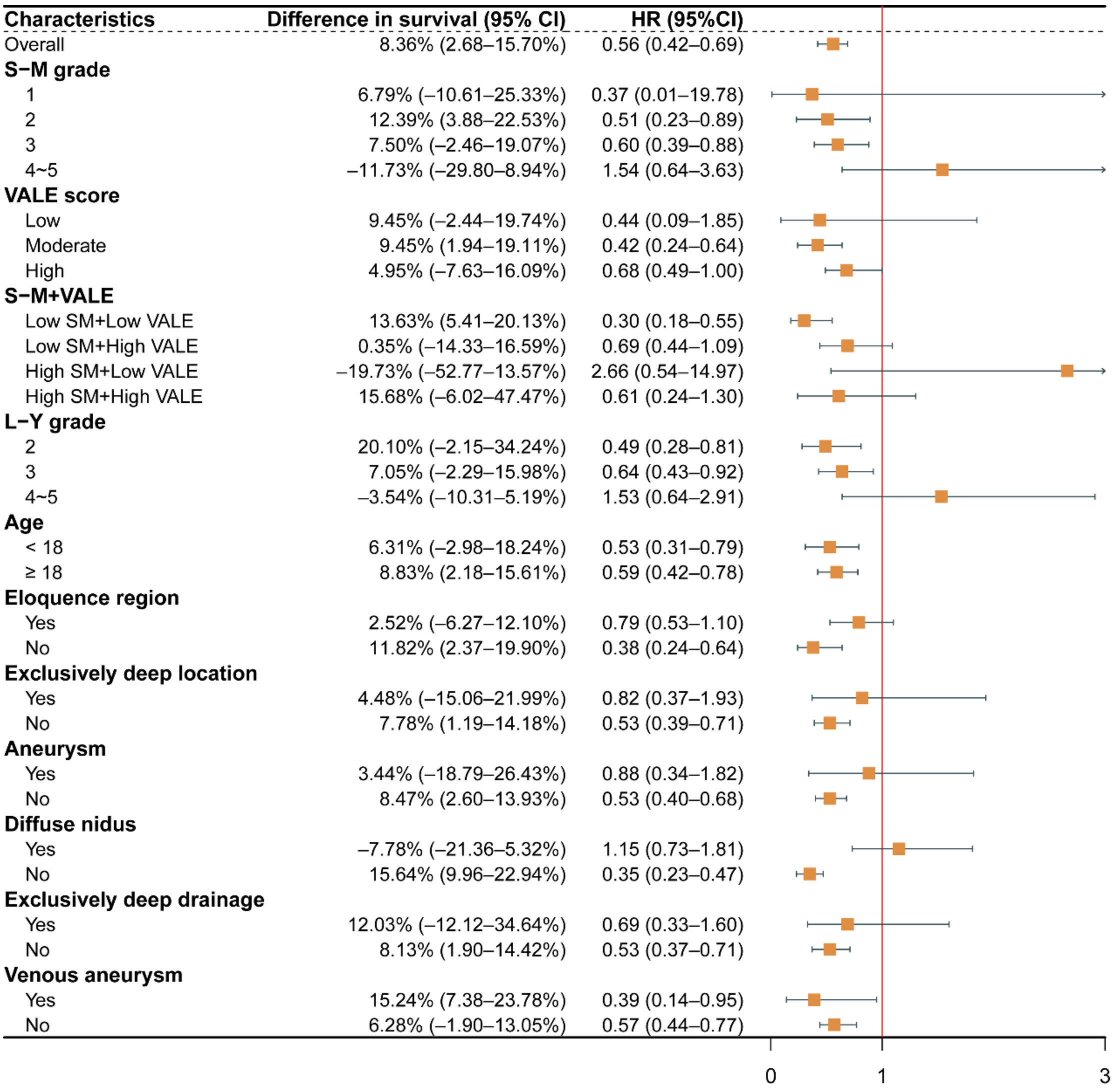

Abbreviations: CI, confidence interval; HR, hazard ratio; L-Y grade, Lawton-Young grade; S-M grade, Spetzler–Martin grade.

© 2025 Han H et al. *JAMA Network Open*.

eTable 1. Protocol of the Hypothetical Target Trial and Emulation

Component	Target trial	Emulated trial
**Aim**	To compare the effect of interventional treatment versus conservative management in patients with unruptured AVMs	Same
**Design**	Multicenter, pragmatic, open-label two-parallel arm superiority randomized trial	Cohort study
**Inclusion**	Patients diagnosed with unruptured AVMs	Same
**Exclusion**	Patients with previous history of interventional treatment towards AVMs	1) Missing essential clinical or imaging data<br>2) Presented with hemorrhage leading to AVM diagnosis.
**Treatment strategies**	1) Conservative management<br>2) Interventional treatment	1) Conservative management within 6 months of diagnosis<br>2) Interventional treatment within 6 months of diagnosis
**Treatment assignment**	Patients are randomly assigned to either strategy	Patients are non-randomly assigned to a treatment strategy. Randomization is emulated via the CCW method
**Start of follow-up**	Date of randomization	Date of diagnosis
**Outcome**	Primary: 5-year hemorrhage-free survival;<br>Secondary: 10-year hemorrhage-free survival	Same
**End of follow-up**	Outcome occurrence, loss to follow up, administrative censoring	Outcome occurrence, deviation from the assigned strategy, loss to follow up, administrative censoring
**Causal contrast**	Intention-to-treat effect and<br>Per protocol effect	Per protocol effect [a]
**Statistical analysis**	Kaplan–Meier survival analysis and Cox proportional hazards models	IPCW-weighted Kaplan–Meier survival analysis and time-dependent Cox proportional hazard models; nonparametric bootstrap to derive 95% CI
**Estimands**	Differences in five/ten-year survival, and hazard ratios	Same

Abbreviations: AVM, arteriovenous malformation; CCW, clone-censor-weight; CI, confidence interval; IPCW, inverse probability of censoring weight.

[a] An intention-to-treat effect was not emulated, as the dataset lacked information on treatment intent at baseline.

© 2025 Han H et al. *JAMA Network Open*.

eTable 2. Breakdown of Missing Data According to Receipt of Actual Treatment

	Overall, No. (%)	Conservative management, No. (%)	Interventional treatment, No. (%)	P
Total	1770	628	1142	
Sex	-	-	-	-
Age at diagnosis	-	-	-	-
Clinical presentation				
Asymptomatic	-	-	-	-
Seizure	-	-	-	-
Headache	-	-	-	-
Neurological deficit	-	-	-	-
Spetzler-Martin grade	17 (1.0)	3 (0.5)	14 (1.2)	0.197
Lawton-Young grade	20 (1.1)	3 (0.5)	17 (1.5)	0.091
VALE score	28 (1.6)	7 (1.1)	21 (1.8)	0.332
Location	-	-	-	-
Eloquent region	-	-	-	-
Size	-	-	-	-
Feeding artery				
Feeding artery dilatation	27 (1.5)	11 (1.8)	16 (1.4)	0.709
Single feeding artery	32 (1.8)	12 (1.9)	20 (1.8)	0.956
Multiple-source supply	22 (1.2)	5 (0.8)	17 (1.5)	0.301
Deep perforator supply	17 (1.0)	1 (0.2)	16 (1.4)	0.021
Feeding artery aneurysm	27 (1.5)	11 (1.8)	16 (1.4)	0.709
Nidus				
Diffuse nidus	20 (1.1)	3 (0.5)	17 (1.5)	0.091
Drainage vein				
Exclusively deep drainage	21 (1.2)	4 (0.6)	17 (1.5)	0.176
Single vein drainage	28 (1.6)	4 (0.6)	14 (1.2)	0.350
Venous stenosis	22 (1.2)	3 (0.5)	19 (1.7)	0.054
Vein aneurysm	22 (1.2)	6 (1.0)	16 (1.4)	0.558

© 2025 Han H et al. *JAMA Network Open*.

eTable 3. Baseline Demographic and AVM Characteristics According to Receipt of Actual Treatment

	Conservative management No. (%)	Interventional treatment No. (%)	P
Total	628	1142	
Sex			0.024
Male	353 (56.2)	706 (61.8)	
Female	275 (43.8)	436 (38.2)	
Age at diagnosis, median (IQR)	25.17 [14.63, 36.71]	27.05 [17.39, 37.88]	0.004
Clinical presentation			
Asymptomatic	49 (7.8)	92 (8.1)	0.923
Seizure	239 (38.1)	488 (42.7)	0.063
Headache	213 (33.9)	356 (31.2)	0.259
Neurological deficit	144 (22.9)	195 (17.1)	0.003
Spetzler-Martin grade			<0.001
1	72 (11.5)	181 (15.8)	
2	165 (26.3)	389 (34.1)	
3	211 (33.6)	384 (33.6)	
4~5	180 (28.7)	188 (16.5)	
Lawton-Young grade			0.016
2	129 (20.5)	242 (21.2)	
3	252 (40.1)	526 (46.1)	
4~5	247 (39.3)	374 (32.7)	
VALE score			0.078
Low risk (–4~–3)	103 (16.4)	212 (18.6)	
Moderate risk (–2~1)	338 (53.8)	645 (56.5)	
High risk (2~5)	187 (29.8)	285 (25.0)	
Location			
Ventricular system involvement	257 (40.9)	386 (33.8)	0.003
Frontal	174 (27.7)	364 (31.9)	0.077
Parietal	208 (33.1)	319 (27.9)	0.026
Temporal	192 (30.6)	327 (28.6)	0.422
Occipital	150 (23.9)	240 (21.0)	0.182
Basal ganglia	58 (9.2)	69 (6.0)	0.017
Thalamus	28 (4.5)	37 (3.2)	0.241
Cerebellum	54 (8.6)	70 (6.1)	0.064
Brainstem	23 (3.7)	26 (2.3)	0.121
Exclusively deep location	110 (17.5)	167 (14.6)	0.125
Infratentorial location	68 (10.8)	91 (8.0)	0.054
Eloquent region	371 (59.1)	577 (50.5)	0.001
Size			<0.001
<3 cm	179 (28.5)	416 (36.4)	
3~6 cm	310 (49.4)	590 (51.7)	
>6 cm	139 (22.1)	136 (11.9)	

© 2025 Han H et al. *JAMA Network Open*.

	Conservative management No. (%)	Interventional treatment No. (%)	P
Feeding artery			
Feeding artery dilatation	415 (66.1)	730 (63.9)	0.391
Single feeding artery	92 (14.6)	209 (18.3)	0.059
Multiple-source supply	261 (41.6)	395 (34.6)	0.004
Deep perforator supply	247 (39.3)	311 (27.2)	<0.001
Feeding artery aneurysm	106 (16.9)	158 (13.8)	0.099
Nidus			
Diffuse nidus	202 (32.2)	220 (19.3)	<0.001
Drainage vein			
Exclusively deep drainage	72 (11.5)	126 (11.0)	0.844
Single vein drainage	232 (36.9)	510 (44.7)	0.002
Venous stenosis	81 (12.9)	126 (11.0)	0.275
Vein aneurysm	192 (30.6)	342 (29.9)	0.826

Abbreviations: AVM, arteriovenous malformation; IQR, interquartile range.

eTable 4. Detailed Treatment Distribution and Follow-Up in the Intervention and Conservative Management Groups

Group	Detailed treatment	5-year outcome			10-year outcome		
		No. (%)	Lost to follow-up (%)	Follow-up years median (IQR)	No. (%)	Lost to follow-up (%)	Follow-up years median (IQR)
Interventional treatment	Overall	539 (100.0%)	51 (9.5)	5.0 [2.6, 5.0]	539	51	5.4 [2.6, 8.8]
	Hemorrhage or lost to follow-up within 6m	33 (6.1)	0 (0.0)	0.2 [0.1, 0.3]	33 (6.1)	0 (0.0)	0.2 [0.1, 0.3]
	Microsurgery only	195 (36.2)	25 (12.8)	5.0 [3.4, 5.0]	195 (36.2)	25 (12.8)	5.3 [3.4, 8.0]
	SRS only	101 (18.7)	17 (16.8)	5.0 [5.0, 5.0]	97 (18.0)	17 (17.5)	8.1 [4.9, 10.0]
	Embolization only	107 (19.9)	9 (8.4)	5.0 [2.2, 5.0]	100 (18.6)	9 (9.0)	5.0 [2.0, 10.0]
	Microsurgery+SRS[a]	3 (0.6)	0 (0.0)	5.0 [5.0, 5.0]	4 (0.7)	0 (0.0)	7.2 [5.8, 8.8]
	Microsurgery+Embolization[a]	11 (2.0)	0 (0.0)	5.0 [2.6, 5.0]	12 (2.2)	0 (0.0)	4.6 [2.9, 5.1]
	SRS+Embolization[a]	87 (16.1)	0 (0.0)	4.5 [2.9, 4.8]	95 (17.6)	0 (0.0)	5.8 [2.71, 9.5]
	Microsurgery+SRS+Embolization[a]	2 (0.4)	0 (0.0)	5.0 [5.0, 5.0]	3 (0.6)	0 (0.0)	8.0 [7.6, 8.7]
Conservative treatment	Overall	1264 (100.0)	0 (0.0)	2.1 [0.6, 5.0]	1264 (100.0)	0 (0.0)	2.1 [0.6, 6.5]
	Hemorrhage or lost to follow-up within 6m	33 (2.6)	0 (0.0)	0.2 [0.1, 0.3]	33 (2.6)	0 (0.0)	0.2 [0.1, 0.3]
	Conservation only	595 (47.1)	0 (0.0)	5.0 [3.3, 5.0]	524 (41.5)	0 (0.0)	6.9 [3.0, 10.0]
	Receiving intervention after 6m	636 (50.3)	0 (0.0)	0.7 [0.6, 2.0]	707 (55.9)	0 (0.0)	0.8 [0.6, 3.0]

Abbreviations: IQR, interquartile range; SRS, stereotactic radiosurgery.

[a] The multimodal treatments are reported by modality combination, irrespective of treatment sequence.

© 2025 Han H et al. *JAMA Network Open*.

eTable 5. Baseline Characteristics of Patients Treated With and Without Microsurgery in the Intervention Group

	Treatment without Microsurgery No. (%)	Treatment with Microsurgery No. (%)	P
Total	328	211	
Female	141 (43.0)	75 (35.5)	0.103
Age at diagnosis, median (IQR)	27.9 [16.7, 37.0]	26.9 [17.0, 37.9]	0.643
Clinical presentation			
Asymptomatic	31 (9.5)	20 (9.5)	>0.999
Seizure	116 (35.4)	102 (48.3)	0.004
Headache	83 (25.3)	55 (26.1)	0.923
Neurological deficit	79 (24.1)	27 (12.8)	0.002
Spetzler-Martin grade			<0.001
1	49 (14.9)	40 (19.0)	
2	84 (25.6)	81 (38.4)	
3	126 (38.4)	75 (35.5)	
4~5	69 (21.0)	15 (7.1)	
Lawton-Young grade			0.836
2	67 (20.4)	43 (20.4)	
3	154 (47.0)	104 (49.3)	
4~5	107 (32.6)	64 (30.3)	
VALE score			<0.001
Low risk (–4~–3)	51 (15.5)	48 (22.7)	
Moderate risk (–2~1)	159 (48.5)	128 (60.7)	
High risk (2~5)	118 (36.0)	35 (16.6)	
Location			
Ventricular system involvement	151 (46.0)	46 (21.8)	<0.001
Frontal	88 (26.8)	98 (46.4)	<0.001
Parietal	113 (34.5)	45 (21.3)	0.002
Temporal	81 (24.7)	69 (32.7)	0.054
Occipital	55 (16.8)	34 (16.1)	0.936
Basal ganglia	37 (11.3)	2 (0.9)	<0.001
Thalamus	16 (4.9)	0 (0.0)	0.003
Cerebellum	25 (7.6)	11 (5.2)	0.359
Brainstem	13 (4.0)	2 (0.9)	0.070
Exclusively deep location	71 (21.6)	13 (6.2)	<0.001
Infratentorial location	35 (10.7)	12 (5.7)	0.065
Eloquent region	205 (62.5)	83 (39.3)	<0.001
Size			0.013
<3 cm	136 (41.5)	77 (36.5)	
3~6 cm	148 (45.1)	119 (56.4)	
>6 cm	44 (13.4)	15 (7.1)	

© 2025 Han H et al. *JAMA Network Open*.

Feeding artery	Treatment without Microsurgery No. (%)	Treatment with Microsurgery No. (%)	P
Feeding artery dilatation	198 (60.4)	135 (64.0)	0.452
Single feeding artery	56 (17.1)	36 (17.1)	>0.999
Multiple-source supply	113 (34.5)	59 (28.0)	0.138
Deep perforator supply	115 (35.1)	35 (16.6)	<0.001
Feeding artery aneurysm	45 (13.7)	22 (10.4)	0.319
Nidus			
Diffuse nidus	74 (22.6)	36 (17.1)	0.151
Drainage vein			
Exclusively deep drainage	58 (17.7)	11 (5.2)	<0.001
Single vein drainage	150 (45.7)	96 (45.5)	>0.999
Venous stenosis	36 (11.0)	24 (11.4)	0.997
Vein aneurysm	95 (29.0)	64 (30.3)	0.808

Abbreviations: IQR, interquartile range.

© 2025 Han H et al. *JAMA Network Open*.

eTable 6. Results for Sensitivity Analyses

	5 years		10 years	
	**Difference in survival (95% CI)[a]**	**Hazard ratio (95% CI)[a]**	**Difference in survival (95% CI)[a]**	**Hazard ratio (95% CI)[a]**
**Main analysis**	7.23% (4.78–9.91%)	0.44 (0.33–0.57)	8.37% (2.68–15.70%)	0.56 (0.42–0.69)
**Sensitivity analysis 1: Alteration of the grace period**				
Grace period of 3 months	7.08% (4.47–9.62%)	0.39 (0.26–0.56)	6.11% (–0.07–14.03%)	0.59 (0.42–0.77)
Grace period of 1 year	6.72% (4.32–9.25%)	0.49 (0.38–0.60)	8.93% (4.05–14.75%)	0.57 (0.46–0.68)
**Sensitivity analysis 2: Separately evaluate the detailed interventional strategies**				
Microsurgery vs conservative	8.72% (5.61–11.53%)	0.20 (0.08–0.62)	18.77% (11.69–25.16%)	0.19 (0.08–0.51)
SRS vs conservative	6.86% (3.05–10.58%)	0.41 (0.18–1.25)	8.06% (0.25–16.69%)	0.58 (0.34–1.00)
Embolization vs conservative	1.94% (–3.90–8.15%)	0.79 (0.40–1.63)	9.71% (–2.00–19.96%)	0.63 (0.32–1.45)
**Sensitivity analysis 3: Changing the eligibility criteria**				
ARUBA-eligible patients	4.97% (1.77–8.55%)	0.54 (0.31–0.79)	8.57% (2.15–15.77%)	0.53 (0.34–0.76)
**Sensitivity analysis 4: Excluding patients with missing data**				
Complete-case patients	7.41% (4.84–10.25%)	0.44 (0.32–0.58)	7.93% (2.44–14.68%)	0.57 (0.42–0.70)
**Sensitivity analysis 5: Using the propensity score method**				
PSM method	7.00% (3.23–10.78%)	0.48 (0.32–0.72)	14.87% (8.28–21.46%)	0.42 (0.29–0.59)
**Sensitivity analysis 6: Redefining the outcome to include functional outcome (mRS ≥ 2)**				
Hemorrhage with mRS ≥ 2	4.07% (2.31–5.58%)	0.30 (0.19–0.51)	3.92% (–0.71–9.81%)	0.47 (0.31–0.69)

[a] The estimands are calculated with the conservative group set as the reference. And the 95% CI derived from nonparametric bootstrapping with 200 replicates.

Abbreviations: CI, confidence interval; PSM, propensity score matching; SRS, stereotactic radiosurgery.

© 2025 Han H et al. *JAMA Network Open*.

eTable 7. Results of the Nested Case-Control Analysis for Hemorrhage Predictive Factors

Characteristics	Univariable analysis		Multivariable analysis	
	OR (95% CI)	P	OR (95% CI)	P
Female	1.02 (0.66-1.58)	0.929		
Age at diagnosis, median (IQR)	1.00 (0.99-1.02)	0.539		
Clinical presentation				
Asymptomatic	1.22 (0.56-2.69)	0.615		
Seizure	**0.42 (0.26-0.69)**	**<0.001**	0.61 (0.35-1.08)	0.088
Headache	0.89 (0.56-1.43)	0.636		
Neurological deficit	1.27 (0.77-2.08)	0.349		
**Interventional treatment**	**0.49 (0.30-0.80)**	**0.004**	**0.47 (0.27-0.80)**	**0.006**
Location				
**Ventricular system involvement**	**2.12 (1.37-3.28)**	**0.001**	**2.02 (1.17-3.46)**	**0.011**
Frontal	**0.52 (0.32-0.86)**	**0.010**	0.79 (0.45-1.38)	0.407
Parietal	1.05 (0.65-1.69)	0.845		
Temporal	1.06 (0.66-1.72)	0.805		
Occipital	1.00 (0.58-1.74)	>0.999		
Basal ganglia	1.19 (0.60-2.37)	0.612		
Thalamus	1.18 (0.43-3.24)	0.747		
Cerebellum	**2.50 (1.23-5.11)**	**0.012**	2.34 (0.86-6.36)	0.096
Brainstem	2.05 (0.75-5.58)	0.160		
Exclusively deep location	**1.94 (1.17-3.20)**	**0.010**	0.64 (0.29-1.43)	0.274
Infratentorial location	1.88 (0.97-3.64)	0.063		
Eloquent region	1.08 (0.70-1.65)	0.728		
Size				
<3 cm	1.00 [Reference]	[Reference]		
3~6 cm	0.77 (0.49-1.22)	0.268		
>6 cm	0.52 (0.26-1.05)	0.069		
Feeding artery				
Feeding artery dilatation	0.65 (0.42-1.00)	0.051		
Single feeding artery	1.45 (0.83-2.52)	0.190		
Multiple-source supply	0.72 (0.44-1.15)	0.166		
Deep perforator supply	**1.69 (1.09-2.62)**	**0.019**	1.24 (0.73-2.12)	0.427
Feeding artery aneurysm	0.86 (0.46-1.59)	0.627		
Nidus				
Diffuse nidus	1.49 (0.95-2.35)	0.083		
Drainage vein				
Exclusively deep drainage	**2.72 (1.53-4.86)**	**0.001**	2.15 (0.97-4.76)	0.058
Single vein drainage	1.23 (0.80-1.90)	0.349		
**Venous stenosis**	**2.77 (1.60-4.81)**	**0.000**	**2.61 (1.38-4.95)**	**0.003**
**Vein aneurysm**	**0.38 (0.20-0.70)**	**0.002**	**0.39 (0.20-0.75)**	**0.005**

Abbreviations: CI, confidence interval; OR, odds ratio.
