# Supplementary material for: Interventional Treatment vs Conservative Management of Unruptured Brain Arteriovenous Malformations
Source: JAMA Netw Open. 2025 Nov 13;8(11):e2543408. doi: 10.1001/jamanetworkopen.2025.43408 (PMC12616460; doi:10.1001/jamanetworkopen.2025.43408)
Supplement: Supplement 2. — Nonauthor Collaborators [file jamanetwopen-e2543408-s002.pdf]

Supplemental Online Content: Nonauthor Collaborators
*First name, last name, and suffix (if applicable) are required and will appear in PubMed.

*Group Name(s): Multimodality Treatment for Brain Arteriovenous Malformation in Mainland China (MATCH) Registry							
***First Name and Middle Initial(s)**	***Last Name**	***Suffix (eg, Jr, III)**	Academic Degrees	Institution	Location (city, state/province, country)	Role or Contribution, eg, chair, principal investigator	Group (if more than 1 Group listed in the byline) and/or Subgroup (eg, Steering Committee)
Qiang	Hao	-	MD	Beijing Tiantan Hospital	Beijing, China	Acquisition of data	Sub-investigator
Hao	Wang	-	MD	Beijing Tiantan Hospital	Beijing, China	Acquisition of data	Sub-investigator
Shuai	Kang	-	MD	Beijing Tiantan Hospital	Beijing, China	Acquisition of data	Sub-investigator
Xun	Ye	-	MD	Beijing Tiantan Hospital	Beijing, China	Acquisition of data	Sub-investigator
Yahui	Zhao	-	MD	Beijing Tiantan Hospital	Beijing, China	Acquisition of data	Sub-investigator
Fa	Lin	-	MD	Beijing Tiantan Hospital	Beijing, China	Acquisition of data	Sub-investigator
Runting	Li	-	MD	Beijing Tiantan Hospital	Beijing, China	Acquisition of data	Sub-investigator
Ke	Wang	-	MD	Beijing Tiantan Hospital	Beijing, China	Acquisition of data	Sub-investigator
Yongenbo	Su	-	MD	Beijing Tiantan Hospital	Beijing, China	Acquisition of data	Sub-investigator
Cunyang	Li	-	MD	Beijing Tiantan Hospital	Beijing, China	Acquisition of data	Sub-investigator
Weitao	Jin	-	MD	Peking University International Hospital	Beijing, China	Acquisition of data	Sub-investigator
Changyu	Lu	-	MD	Peking University International Hospital	Beijing, China	Acquisition of data	Sub-investigator
Yang	Zhao	-	MD	Peking University International Hospital	Beijing, China	Acquisition of data	Sub-investigator
Yunfan	Zhou	-	MD	Peking Union Medical College Hospital	Beijing, China	Acquisition of data	Sub-investigator
Geng	Guo	-	MD	First Hospital of Shanxi Medical University	Shanxi, China	Acquisition of data	Sub-investigator
Xiangyu	Meng	-	MD	The First Hospital of Hebei Medical University	Hebei, China	Acquisition of data	Sub-investigator
Jun	Pu	-	MD	The second Affiliated Hospital of Kunming Medical University	Yunnan, China	Acquisition of data	Sub-investigator
Zhiyong	Shi	-	MD	Nanjing Drum Tower Hospital	Jiangsu, China	Acquisition of data	Sub-investigator
Qiang	Wang	-	MD	The Second Affiliated Hospital of Xuzhou Medical University	Jiangsu, China	Acquisition of data	Sub-investigator
Junlin	Lu	-	MD	West China Hospital	Sichuan, China	Acquisition of data	Sub-investigator

Supplemental Online Content: Nonauthor Collaborators
*First name, last name, and suffix (if applicable) are required and will appear in PubMed.

***First Name and Middle Initial(s)**	***Last Name**	***Suffix (eg, Jr, III)**	Academic Degrees	Institution	Location (city, state/province, country)	Role or Contribution, eg, chair, principal investigator	Group (if more than 1 Group listed in the byline) and/or Subgroup (eg, Steering Committee)
Weiwei	Chen	-	MD	The First Affiliated Hospital of Anhui Medical University	Anhui, China	Acquisition of data	Sub-investigator
