# Supplementary material for: Interventional Treatment vs Conservative Management of Unruptured Brain Arteriovenous Malformations
Source: JAMA Netw Open. 2025 Nov 13;8(11):e2543408. doi: 10.1001/jamanetworkopen.2025.43408 (PMC12616460; doi:10.1001/jamanetworkopen.2025.43408)
Supplement: Supplement 3. — Data Sharing Statement [file jamanetwopen-e2543408-s003.pdf]

Data Sharing Statement

Han. Interventional Treatment vs Conservative Management of Unruptured Brain Arteriovenous Malformations. *JAMA Netw Open*. Published November 13, 2025. doi:10.1001/jamanetworkopen.2025.43408

Data
Data available: Yes
Data types: Deidentified participant data
How to access data: The de-identified participant data and accompanying data dictionary used in this analysis will be accessible upon reasonable request, provided that the requester signs the necessary data-sharing agreements.
When available: With publication

Supporting Documents
Document types: Statistical/analytic code
How to access documents: Data access inquiries should be directed to the corresponding authors. Approval from relevant ethics committees and data custodians is required for all requests.
When available: With publication

Additional Information
Who can access the data: Anyone requesting the data, researchers whose proposed use of the data has been approved.
Types of analyses: For any purpose.
Mechanisms of data availability: Approval from relevant ethics committees and data custodians is required for all requests.
